# Paludification reduces black spruce growth rate but does not alter tree water use efficiency in Canadian boreal forested peatlands

**DOI:** 10.1186/s40663-021-00307-x

**Published:** 2021-05-12

**Authors:** Joannie Beaulne, Étienne Boucher, Michelle Garneau, Gabriel Magnan

**Affiliations:** 1 Geotop Research Center, Université du Québec à Montréal, Montréal, Québec H3C 3P8 Canada; 2 Department of Geography, Université du Québec à Montréal, Montréal, Québec H3C 3P8 Canada; 3 GRIL-UQAM, Université du Québec à Montréal, Montréal, Québec H3C 3P8 Canada; 4 Centre d’études nordiques, Université Laval, Montréal, Québec G1V 0A6 Canada

**Keywords:** Black spruce growth, Boreal biome, Carbon allocation, Ecophysiological mechanisms, Forested peatland, Paludification, Stable isotope, Water use efficiency

## Abstract

**Background:**

Black spruce (*Picea mariana* (Mill.) BSP)-forested peatlands are widespread ecosystems in boreal North America in which peat accumulation, known as the paludification process, has been shown to induce forest growth decline. The continuously evolving environmental conditions (e.g., water table rise, increasing peat thickness) in paludified forests may require tree growth mechanism adjustments over time. In this study, we investigate tree ecophysiological mechanisms along a paludification gradient in a boreal forested peatland of eastern Canada by combining peat-based and tree-ring analyses. Carbon and oxygen stable isotopes in tree rings are used to document changes in carbon assimilation rates, stomatal conductance, and water use efficiency. In addition, paleohydrological analyses are performed to evaluate the dynamical ecophysiological adjustments of black spruce trees to site-specific water table variations.

**Results:**

Increasing peat accumulation considerably impacts forest growth, but no significant differences in tree water use efficiency (iWUE) are found between the study sites. Tree-ring isotopic analysis indicates no iWUE decrease over the last 100 years, but rather an important increase at each site up to the 1980s, before iWUE stabilized. Surprisingly, inferred basal area increments do not reflect such trends. Therefore, iWUE variations do not reflect tree ecophysiological adjustments required by changes in growing conditions. Local water table variations induce no changes in ecophysiological mechanisms, but a synchronous shift in iWUE is observed at all sites in the mid-1980s.

**Conclusions:**

Our study shows that paludification induces black spruce growth decline without altering tree water use efficiency in boreal forested peatlands. These findings highlight that failing to account for paludification-related carbon use and allocation could result in the overestimation of aboveground biomass production in paludified sites. Further research on carbon allocation strategies is of utmost importance to understand the carbon sink capacity of these widespread ecosystems in the context of climate change, and to make appropriate forest management decisions in the boreal biome.

**Supplementary Information:**

The online version contains supplementary material available at 10.1186/s40663-021-00307-x.

## Background

Black-spruce (*Picea mariana* (Mill.) BSP)-dominated forested peatlands are widespread ecosystems in boreal North America (Korhola 1995; Crawford et al. 2003; Lavoie et al. 2005). In such environments, *Sphagnum* moss growth is favored and leads to the development of thick organic layers that maintain cool, acid, humid, and anaerobic soil conditions (van Cleve et al. 1983; Fenton and Bergeron 2006). As this paludification process progresses, soil temperature and nutrients become limiting, and forest growth eventually declines (Boudreault et al. 2002; Harper et al. 2003; Simard et al. 2007; Lafleur et al. 2011), leading to tree dieback and an opening of forest stands. This negatively impacts the forest productivity and, therefore, several management practices have been developed to reduce or even reverse this process (e.g., Lavoie et al. 2005; Bergeron et al. 2007; Fenton et al. 2009). However, in order to enhance our capacity to anticipate the effects of paludification on forest productivity, there is a need to shed light on the ecophysiological mechanisms that control forest growth decline in paludified ecosystems.

Based on existing ecophysiological theory (mostly developed in non-paludified sites), three mechanisms may possibly be invoked either jointly or separately to explain such declines in black spruce growth. First, paludification process may reduce carbon assimilation rates (*A*), as it is well known that the photosynthesis apparatus of plants is sensitive to thermal (Göbel et al. 2019), oxygen (Bartholomeus et al. 2008) or nutrient limitations (Longstreth and Nobel 1980). A reduction in *A* would imply the downregulation of gross primary production (GPP) and less photosynthates export to stem growth. In temperate environments, such a positive relationship between GPP and stem growth has been elucidated (Belmecheri et al. 2014), but this relationship is far from being applicable to boreal and paludified forests. In boreal environments, relationships between carbon uptake and forest growth are either inconclusive (Rocha et al. 2006) or decoupled (Pappas et al. 2020), while remaining unexplored in paludified ecosystems. Second, growth decline could also result from a reduction of stomatal conductance (*g*_s_). When trees are severely affected by drought stress, stomatal closure allows plants to reduce water losses during transpiration, but this mechanism also penalizes carbon uptake and ultimately results in lower growth rates (Linares and Camarero 2012). However, in paludified environments, adjustments of plant stomata to peat accumulation, climate variability, and water table variations are yet to be determined. Lastly, trees growing in nutrient-poor/low oxygen settings are more susceptible to allocate carbon to the root system rather than to aboveground components (Giardina et al. 2003; Vicca et al. 2012). Thus, changes in carbon allocation strategies favoring belowground biomass as peat accumulates could explain the apparent growth decrease visible in stems.

Another source of uncertainty arises from the fact that trees growing in paludified ecosystems deal with continuously evolving environmental conditions. The accumulation of thick organic layers induces significant changes that highlight the dynamic interactions occurring in boreal forested peatlands. For example, paludification is characterized by water table rise (Lavoie et al. 2005; Fenton and Bergeron 2006), and interactions over time may exist between water table fluctuations, stem growth, and tree water use efficiency (i.e., the ratio of carbon assimilated to water losses through evapotranspiration; Farquhar et al. 1989). These interactions, which remain largely unexplored, may vary in function of the degree of paludification (i.e., organic layer thickness). Consequently, how ecophysiological mechanisms adjust to such changes and how they impact tree radial growth over time needs to be addressed.

Carbon and oxygen stable isotopes in tree-ring cellulose may be of great help to disentangle ecophysiological processes responsible for tree growth decline in paludified environments. Indeed, gas exchange dynamics at the leaf-atmosphere interface are imprinted in the isotopic signature of annually-produced wood. For example, both *A* and *g*_s_ affect carbon stable isotope fractionation in tree-ring cellulose (Scheidegger et al. 2000; Cernusak et al. 2013), and the ratio between both, namely intrinsic water use efficiency (iWUE = *A*/*g*_s_), is at the core of ecosystem functioning (Guerrieri et al. 2019). Moreover, fractionation of oxygen stable isotopes reflects the magnitude of stomatal controls (through *g*_s_) on transpiration jointly with the signal of source water uptake (Barbour 2007). Tree-ring based analysis of ^13^C and ^18^O can help tracking dynamical adjustments of ecophysiological parameters to changing environmental conditions (Voelker et al. 2016). Such strategies have been well studied for well-drained environments (Frank et al. 2015; Voelker et al. 2016), but have never been investigated for paludified sites. In addition, the use of a paleoecological approach may be useful to investigate the interactions between tree growth mechanisms and local environmental conditions in boreal forested peatlands. Analyses of peat cores are commonly used to document, among others, past changes in both vegetation composition and hydrological conditions. For example, testate amoeba (unicellular protists) shells preserved in peat are widely used proxies for reconstructing variations in water table depths (Mitchell et al. 2008). Such paleoecological and paleohydrological analyses have previously been conducted in forested peatlands (Ruppel et al. 2013; Le Stum-Boivin et al. 2019; Magnan et al. 2019), but have never been put in relation to tree growth.

In this study, we investigate the mechanisms that drive tree growth decline in black-spruce-dominated forested peatlands of eastern Canada. To do this, we compare tree-ring-derived growth trends and stable-isotope-inferred ecophysiological processes along a paludification gradient characterized by increasingly thicker peat deposits. More specifically, the objectives of the study are to (1) determine whether the decline in stem growth induced by the paludification process is attributable to changes in ecophysiological processes (such as *A* or *g*_s_) and/or to changes in carbon allocation strategies, and (2) evaluate the dynamical ecophysiological adjustments to ever-changing environmental conditions. We hypothesised that (1) growth decline results from a reduction of water use efficiency (iWUE), (2) reductions in iWUE are greater in the most paludified sites, and (3) strongest ecophysiological adaptation also take place in the most paludified sites. In order to test these hypotheses, we used an innovative approach that combines peat-based paleohydrological analyses – to reconstruct past variations in water table depth – and stable isotope analyses in tree rings – to investigate iWUE changes through time.

## Methods

### Study area

**Fig. 1:**
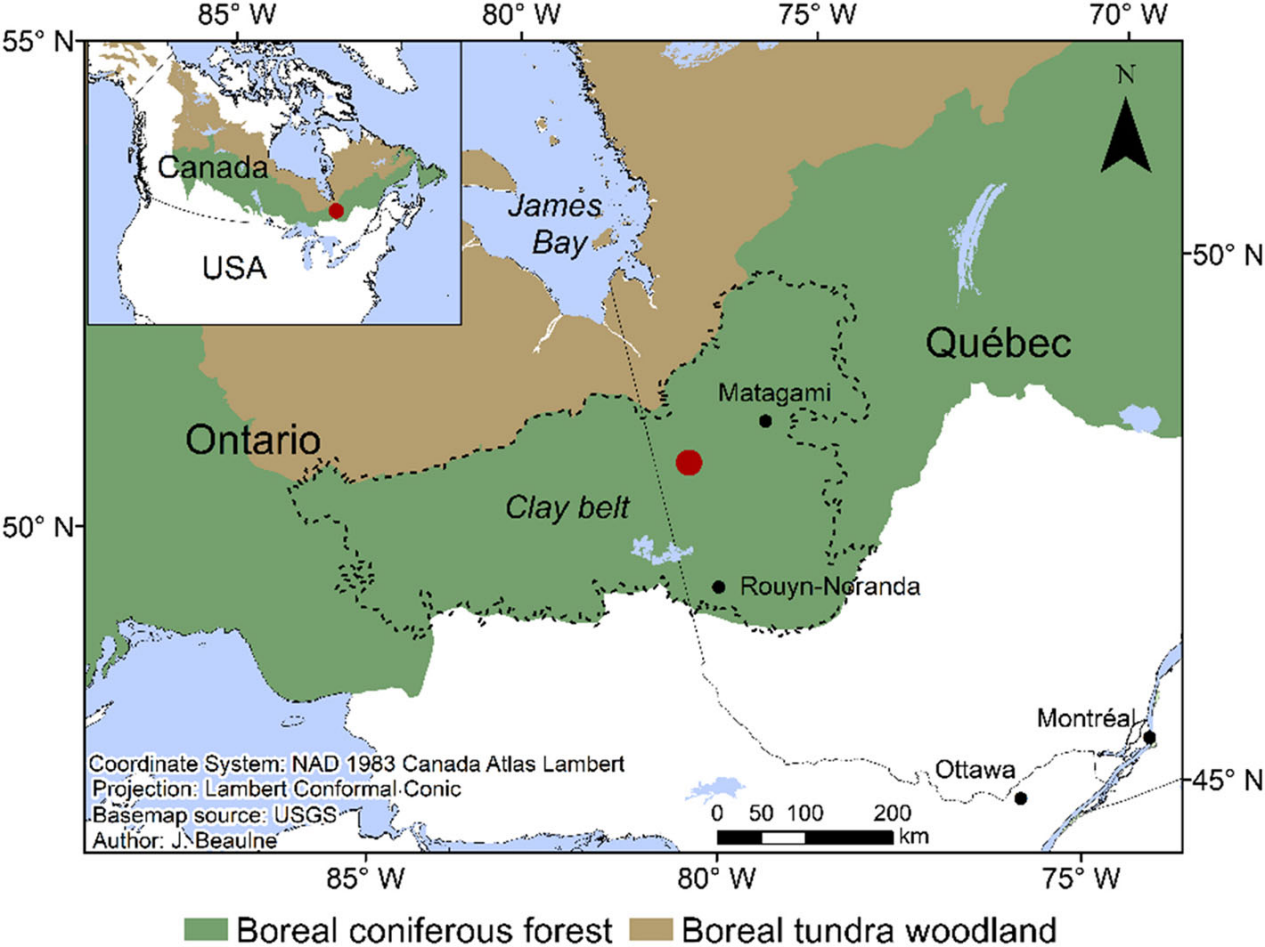
Location of the studied Casa boreal forested peatland (red dot)

The study was conducted in the south of James Bay in eastern Canada, within the Clay Belt region which is part of the black spruce-feather moss bioclimatic domain (Saucier et al. 2009; Fig. 1). This area is particularly prone to paludification due to the relatively cold and humid climate, the flat topography, and the dominance of poorly-drained clayey sediments left by the proglacial lakes Barlow and Ojibway (Vincent and Hardy 1977). Mean annual temperature is 0.3 °C (over the 1950–2013 period), ranging from − 18.9 °C in January to 16.3 °C in July, and mean annual precipitation is 818 mm (McKenney et al. 2011). The regional fire cycle is estimated at ~ 400 years since 1920 (Bergeron et al. 2004), allowing the accumulation of thick organic layers in forests between fire events.

### Site selection and sampling

The Casa forested peatland (49°33′06″ N, 78°59′10″ W; Fig. S2.1) was selected following the studies of Magnan et al. (2020) and Le Stum-Boivin et al. (2019) due to its regional representativeness in terms of topography, vegetation composition, and canopy openness. The slope is < 1% and the organic layer thickness varies between 40 cm and more than 1 m along the selected transect. The canopy cover is relatively closed but gradually opens up with organic layer thickening, which is typically observed in forested peatlands of the Clay Belt. The aboveground vegetation is largely dominated by black spruce and ericaceous shrubs, such as *Vaccinium angustifolium* Ait., *Rhododendron groenlandicum* (Oeder) Kron and Judd, *Kalmia angustifolia* L., and *Chamaedaphne calyculata* (L.) Moench. The understory is dominated by *Sphagnum* communities, particularly *S. angustifolium* (C. Jens. ex Russ.) C. Jens. and *S. fallax* (Klinggr.) Klinggr. under the tree canopy, and *S. fuscum* (Schimp.) Klinggr. where the canopy cover is more open.

**Fig. 2:**
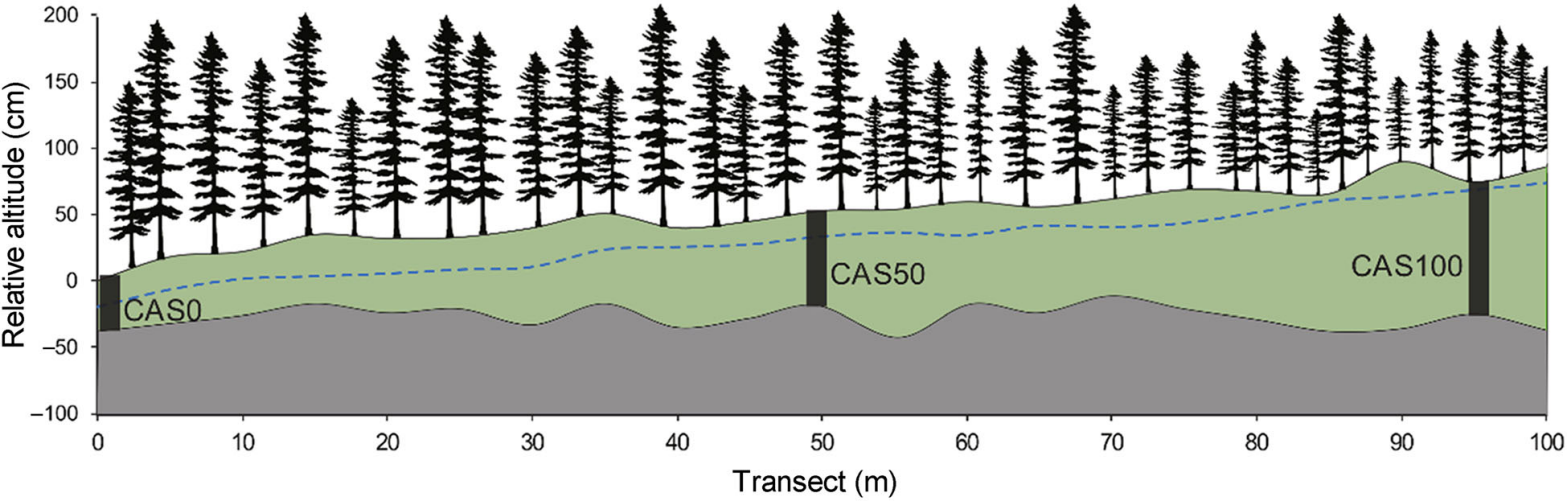
Schematic representation of the three sites along the study transect. Relative altitude of the organic layer (green) and the mineral surface (grey) are shown. Black rectangles represent the location of the sampled peat cores. The dotted blue line indicates the depth of the water table measured in the field. Trees are not to scale but are representative of variations in canopy openness along the transect

Three sampling sites (CAS0, CAS50, CAS100) were established along a 100-m transect following an organic matter thickness gradient within the selected forested peatland (Fig. 2). At each site, one peat monolith was sampled down to the mineral contact using a Box corer (Jeglum et al. 1992). Sampling locations were chosen to be representative of the degree of paludification of each site in terms of peat thickness and canopy opening. Relative surface altitude and peat thickness were measured at 5-m intervals along the transect using a high precision altimeter (ZIPLEVEL PRO-2000) and an Oakfield probe. Water table depths were measured at the same intervals, in June 2017 and September 2018, a few hours after holes were dug to make sure that the water table level had stabilized. Twenty black spruce trees were also sampled at each site within a 10-m radius of the collected peat core. Only dominant and codominant trees with straight stems and no visible scars were selected. Peat thickness was measured at the bottom of each sampled tree to validate the concordance with the mean peat thickness of the site. The diameter at breast height (DBH) and the height of selected trees were measured and cross-sections were collected at standard height (1.3 m). The root system of one black spruce tree per site was excavated to verify the depth of the rooting zone and the growth substrate (i.e., mineral or organic matter). Moreover, tree aboveground biomass of each site was estimated by measuring the diameter at breast height (DBH) of all trees (DBH ≥ 1 cm) within a 10 m × 10 m plot and then using allometric equations adapted to black spruce growth (Ung et al. 2008).

### Black spruce radial growth analysis

The 60 dried cross-sections were finely sanded (from 80 to 600 grit size) prior to ring-width measurements along two radii using CooRecorder software (version 8.1.1; Larson 2016). Samples were visually cross-dated using PAST5 software (version 5.0.610; Knibbe 2019), and skeleton plots were generated using the R package *dplR* (version 1.6.9; Bunn et al. 2018). Ring-width series were converted to annual basal area increment (BAI) to compare tree aboveground productivity between the three sites, as BAI is more representative of three-dimensional stem growth than the linear ring-width measurements (Husch et al. 2003; Biondi and Qeadan 2008). Individual BAI series were produced using the R package *dplR* (version 1.6.9; Bunn et al. 2018), and yearly averages were then calculated using all trees from the same site. Ring-width series were also standardized (Fig. S2.7) in order to perform correlation analyses with climate data (Supplementary Material 1.1).

### Isotopic analysis of tree rings

Black spruce ecophysiological processes were evaluated from carbon (δ^13^C) and oxygen (δ^18^O) isotopic ratio analyses. These were performed on five trees per site and from two wood strips per tree (i.e., a total of 30 samples). Sample preparation was carried out following the protocol described in Giguère-Croteau et al. (2019) (Supplementary Material 1.2). A five-year resolution over a 100-year period (1919–2018) was considered. Alpha-cellulose was extracted, as suggested for black spruce samples (Bégin et al. 2015), following the protocol used by Naulier et al. (2014).

Tree-ring δ^13^C values vary according to discrimination against ^13^C during photosynthesis, defined as (Farquhar et al. 1982):

1 $$ {\Delta }^{13}\mathrm{C}=\frac{\updelta^{13}{\mathrm{C}}_{\mathrm{air}}-{\updelta}^{13}{\mathrm{C}}_{\mathrm{tree}}}{1+\left({\updelta}^{13}{\mathrm{C}}_{\mathrm{tree}}/1000\right)}, $$

where δ^13^C_air_ is the carbon isotope ratio of the atmosphere and δ^13^C_tree_ is the isotopic value of the tree ring. δ^13^C_air_ values were taken from McCarroll and Loader (2004) for the 1919–2003 period, and were linearly extrapolated for the 2004–2018 period. Because of the five-year resolution of δ^13^C_tree_ values, we averaged the δ^13^C_air_ values over 5 y. Following Farquhar et al. (1989), ∆^13^C is related to leaf intercellular CO_2_ concentration (*c*_i_) and ambient CO_2_ concentration (*c*_a_) according to the following equation:

2 $$ {\Delta}^{13}\mathrm{C}=a+\left(b-a\right)\left(\frac{c_{\mathrm{i}}}{c_{\mathrm{a}}}\right), $$

where *a* (4.4‰) is the fractionation occurring during CO_2_ diffusion through stomata (O’Leary 1981) and *b* (27‰) is the fractionation due to carboxylation by the Rubisco enzyme (Farquhar and Richards 1984). Values of *c*_a_ were obtained from the Mauna Loa Observatory (esrl.noaa.gov/gmd/ccgg/). Intrinsic water use efficiency (iWUE), defined as the amount of carbon assimilated per unit of water lost, can then be estimated from *c*_i_ and *c*_a_ as follows (Ehleringer et al. 1993):

3 $$ \mathrm{iWUE}=\left(\frac{A}{g_{\mathrm{s}}}\right)=\left(\frac{c_{\mathrm{a}}-{c}_{\mathrm{i}}}{1.6}\right),\kern0.5em $$

where *A* is the rate of CO_2_ assimilation, *g*_s_ is the stomatal conductance, and the constant 1.6 represents the ratio of water vapor and CO_2_ diffusivity in air. Equation 3 shows that the difference between *c*_a_ and *c*_i_ is related to the ratio of assimilation (*A*) to stomatal conductance (*g*_s_).

Since the δ^18^O composition of tree rings is mainly controlled by leaf water composition and enrichment due to transpiration of lighter oxygen isotopes, δ^18^O values are assumed to be related to the stomatal conductance and independent of photosynthetic activity (Yakir 1992; Barbour 2007). Therefore, by combining δ^13^C and δ^18^O analyses it is possible to discriminate the effects of changes in photosynthetic rate (*A*) and stomatal behavior (*g*_s_) on iWUE (Scheidegger et al. 2000).

### Peat-based paleoecohydrological reconstructions

In order to evaluate the response of black spruce ecophysiological mechanisms to hydrological variations, water table depths were reconstructed from peat core analyses. The collected cores were cut into 1 cm-thick slices before analysing testate amoeba assemblages at 1-cm intervals. Testate amoeba shells were extracted following the standard protocol of Booth et al. (2010) (Supplementary Material 1.3). Samples were then analysed under an optical microscope (400× magnification). A minimum of 100 tests was counted per sample, except in highly humified peat samples, in which test concentration was very low. In these cases, no water table depth (WTD) was inferred, as the total count (< 20 tests) was insufficient to ensure reliable WTD reconstruction (Payne and Mitchell 2009). Past WTDs were reconstructed using a weighted average model with tolerance down-weighting and inverse deshrinking (WA.inv.tol). The transfer function was built using the R package *rioja* (version 0.9–15.1; Juggins 2017), from a modern dataset of 272 surface samples combining non-forested (Lamarre et al. 2013) and forested peatlands (Beaulne et al. 2018 and this study) of eastern Canada. High inferred WTD values corresponded to drier surface conditions.

Plant macrofossils and macroscopic charcoal particles (> 0.5 mm) were also analysed along each peat core to reconstruct vegetation dynamics since peat initiation and better understand the paludification process at each site (Supplementary Material 1.4).

### Peat core chronologies

A total of 11 samples were submitted to A. E. Lalonde AMS Laboratory (University of Ottawa, Canada) for accelerator mass spectrometry radiocarbon dating (^14^C). Plant macrofossil remains were carefully selected to date peat initiation, the last fire event, and main transitions in vegetation composition at each sampling site (Beaulne et al. 2021). The ^14^C dates were calibrated using the IntCal13 calibration curve (Reimer et al. 2013). ^210^Pb dating was also achieved for the uppermost 24–26 cm of peat cores at 1-cm intervals by alpha spectrometry (EGG Ortec 476A) at the GEOTOP Research Center (Université du Québec à Montréal, Canada). Ages were inferred by ^210^Pb activity measurement, using the constant rate of supply model (Appleby and Oldfield 1978) following HNO_3_-HCl-H_2_O_2_ sample digestion (Ali et al. 2008). Age-depth models were generated using the *rbacon* package in R (version 2.3.9.1; Blaauw and Christen 2019). Ages are expressed in calendar years before present (cal yr BP; 1950 CE) and the age of the peat surface is therefore set to − 67 cal yr BP (coring year: 2017 CE).

### Statistical analyses

Statistical analyses were achieved to compare data in function of the degree of paludification. One-way ANOVA analyses were performed to test for differences in BAIs, WTDs, δ^13^C-derived ecophysiological parameters, and tree-ring δ^18^O between sites. Post-hoc Tukey Honest Significant Difference (HSD) were performed to identify significant differences in the mean for each site. Breakpoints in slopes were also estimated from Davies test (Davies 1987) for δ^13^C-derived ecophysiological parameters, tree-ring δ^18^O, and WTDs. All statistical analyses were performed in R (R Core Team 2018).

## Results

**Fig. 3:**
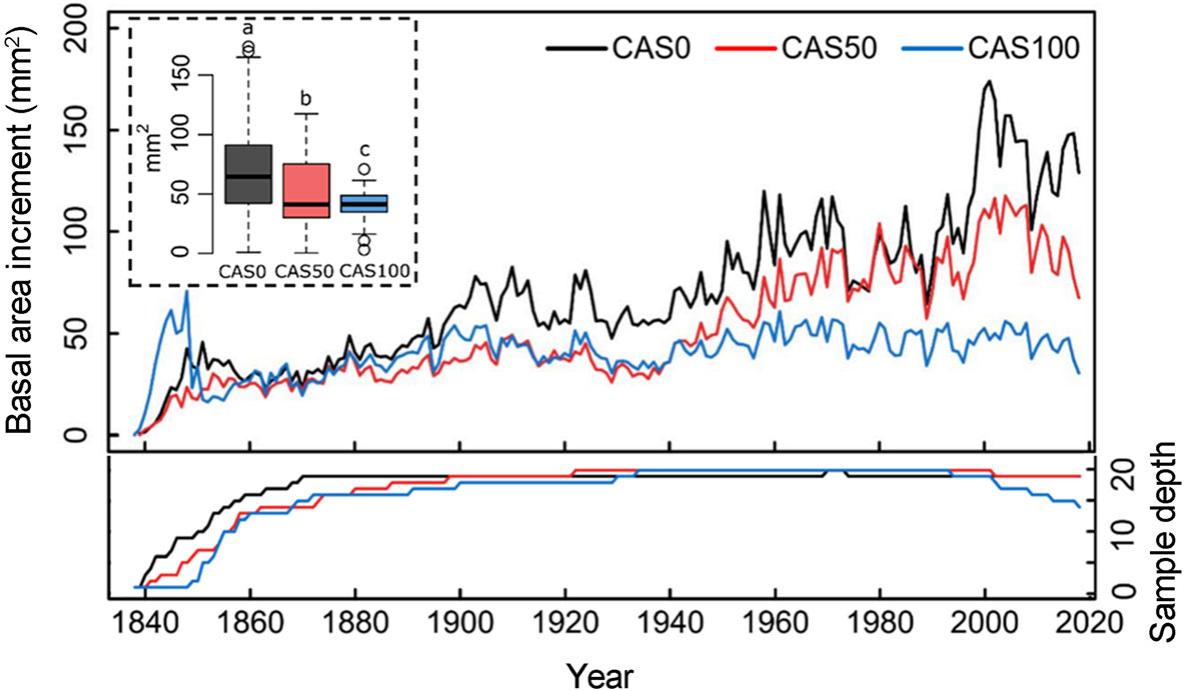
Mean annual basal area increment of black spruce trees since their establishment after the last fire event. The decrease in sample depth (i.e., number of trees included in the chronologies) at CAS100 since 2000 is explained by some trees for which the latest rings were partly missing. See Fig. S2.3 for BAI distribution. Boxplots show the median, quartile range, extreme values (dotted lines), and outliers (dots) of the distribution. Different letters above the boxes indicate significant differences between the sites based on Tukey’s HSD test

The study sites CAS0, CAS50, and CAS100 have an organic layer thickness of 40, 73, and 98 cm, respectively (Table 1). Tree-ring analyses revealed even-aged stands covering the period 1839–2018 CE at each site (see sample depth in Fig. 3 for tree age variability). Radiocarbon dating of the most recent charcoal layer indicates that the last fire event occurred between 0 and 290 cal yr BP (median age: 175–179 cal yr BP; Table S2.1). These results suggest that trees were from the first cohort that grew after the last local fire, which most likely occurred around 200–250 years ago (~ 1800 CE). The depth of the uppermost charcoal layer in the peat profile indicates that black spruce established in a residual organic layer of approximately 15, 45, and 65 cm at sites CAS0, CAS50, and CAS100, respectively. The root system excavation of the three selected trees suggests that roots reached the mineral soil at CAS0 and CAS50, but were restricted to the organic layer at CAS100.

**Table 1:** Characteristics of the three study sites^a^

Site	Mean organic layer thickness (cm)	Core length (cm)	Mean WTD (cm)	Mean DBH^b^ (cm)	Tree aboveground biomass^b^ (kg·m^− 2^)	Tree density^b,c^ (trees·ha^− 1^)	Mean tree height^d^ (m)
CAS0	40 ± 5	38	25 ± 2	10.0 ± 1.6	8.9 ± 0.3	1200	13.8 ± 0.4
CAS50	73 ± 4	69	20 ± 1	9.1 ± 1.1	7.6 ± 0.3	1200	11.3 ± 0.5
CAS100	98 ± 4	95	10 ± 2	5.6 ± 0.8	4.6 ± 0.2	1000	10.4 ± 0.5

^a^ ± values correspond to standard errors

^b^ Calculated from all trees (DBH ≥ 1 cm) within a 10 m × 10 m plot, which includes 20, 24, and 34 trees for sites CAS0, CAS50, and CAS100 respectively

^c^ Include trees with a diameter at breast height (DBH) ≥ 9 cm

^d^ Calculated from the 20 black spruce trees (dominant and co-dominant) sampled at each site

### Black spruce radial growth

Tree height and DBH values decrease along the paludification gradient, with the lowest values observed at the most paludified site where the organic layers are the thickest (Table 1). Mean DBHs of 10.0, 9.1, and 5.6 cm were calculated for CAS0, CAS50, and CAS100, respectively. BAIs also indicate a significant decrease in stem growth rates with increasing peat thickness (Table 2; Fig. 3, Fig. S2.3). Trees from CAS0 added a greater wood surface with age, resulting in an increasing BAI trend (mean BAI = 70 mm^2^). At CAS50, tree radial growth followed similar patterns but was more limited (mean BAI = 51 mm^2^). In contrast, trees from CAS100 maintained relatively constant BAI values, resulting in decreased wood production (mean BAI = 40 mm^2^). Estimates of tree aboveground biomass showed similar trends with values of 8.9, 7.6, and 4.6 kg·m^− 2^ for sites CAS0, CAS50, and CAS100, respectively.

**Table 2:**
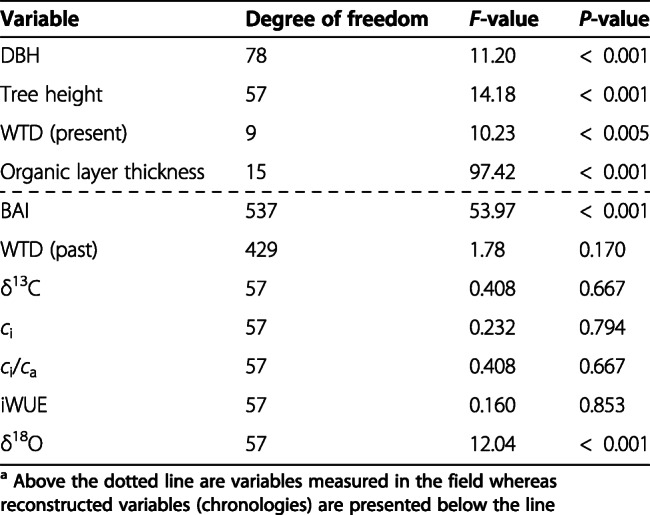
Results of one-way ANOVA analyses comparing the three study sites^a^

### Trends in δ^13^C, δ^18^O, and iWUE

**Fig. 4:**
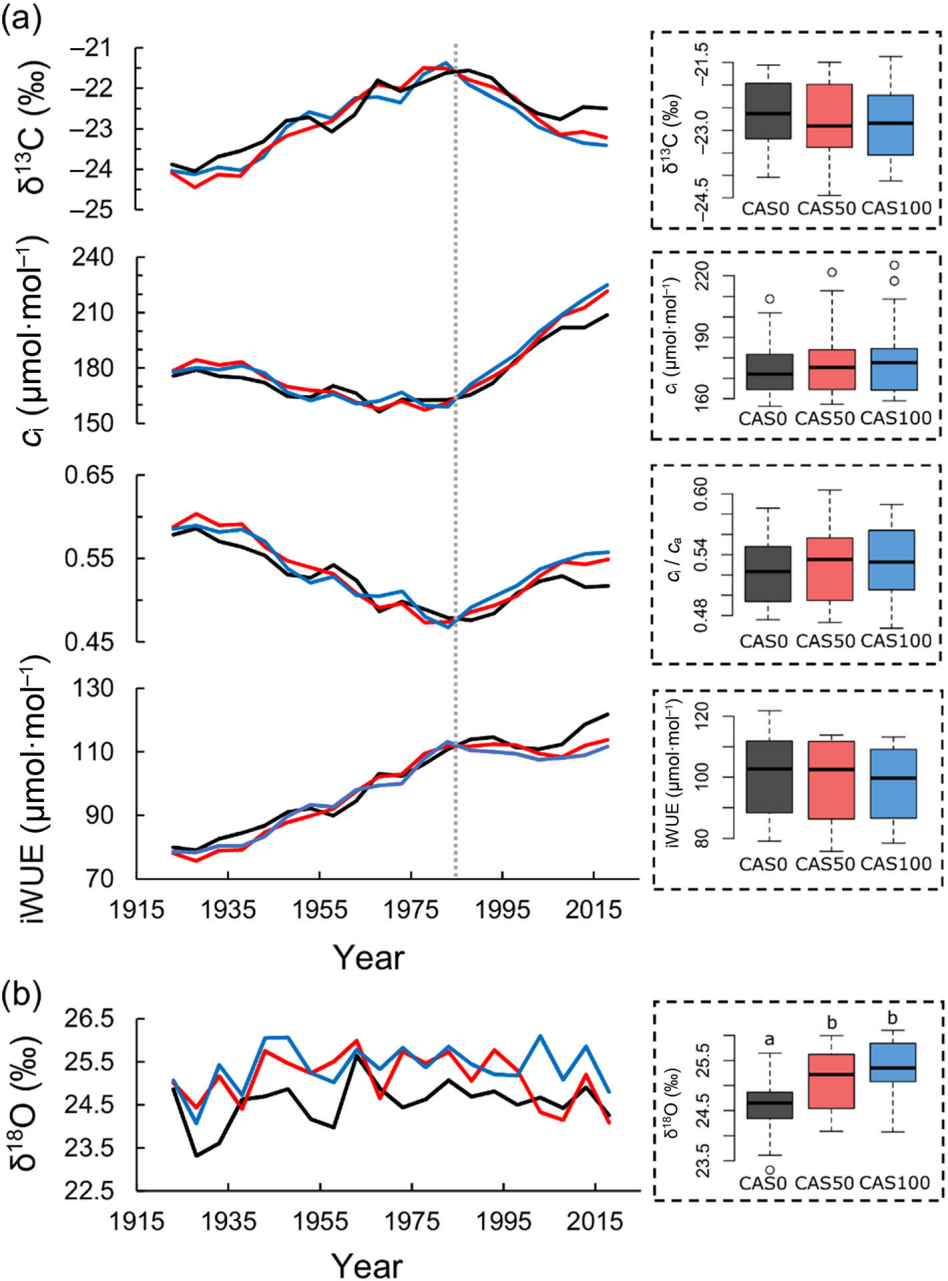
Black spruce ecophysiological response to rising *c*_i_ based on five-year resolution δ^13^C and δ^18^O analyses for the period 1919–2018. **a** Tree-ring δ^13^C and δ^13^C-derived ecophysiological parameter values (*c*_i_, *c*_i_/*c*_a_, iWUE); **b** tree-ring δ^18^O values. Results from CAS0, CAS50, and CAS100 are shown in black, red, and blue, respectively. Boxplots show the median, quartile range, extreme values (dotted lines), and outliers (dots) of the distribution. Different letters above the boxes indicate significant differences between the sites based on Tukey’s HSD test (the absence of letters means no significant difference between the three sites). The grey dotted line represents a significant breakpoint (*P* <  0.01) in slopes based on Davies test

The δ^13^C-derived ecophysiological parameters do not differ between the three sites over the 1919–2000 period (Fig. 4a, Table 2). Over time, black spruce trees used two different strategies in response to rising *c*_a_. A substantial increase in iWUE was first observed until the 1980s (*c*_a_ ≈ 340 ppm), along with relatively stable intercellular CO_2_ concentration (*c*_i_). During this period, iWUE increased by 43% at each site. A major shift in tree ecophysiology then occurred in the mid-1980s as *c*_i_ began to increase considerably. In parallel, iWUE stabilized until 2018, except for at CAS0, where a new increase seems to have begun around 2000. However, this recent trend at CAS0 should be interpreted with caution considering its short duration and the five-year resolution.

Tree-ring cellulose δ^18^O analyses show no significant trends for the three sites across the whole record (Fig. 4 b, Table 2). However, oxygen stable isotope ratios were systematically lower at the least paludified site (CAS0), suggesting a greater depletion in heavy isotopes. For all series, tree-ring δ^18^O values increased until ~ 1950 and became more constant afterwards, except for a slight decrease in the early 2000s at CAS50.

### Paleoecohydrological reconstructions

Both hydrological conditions and vegetation composition were similar between the three sites throughout the duration of black spruce growth (Figs. S2.4, S2.5). Macrofossil analysis showed that the last fire (~ 1800 CE) induced a shift in vegetation composition from high dominance of woody vegetation to a black spruce-*Sphagnum*-dominated stand (Fig. S2.4). The canopy opening allowed rapid *Sphagnum* moss expansion in the bryophyte layer while the black spruce post-fire cohort established.

**Fig. 5:**
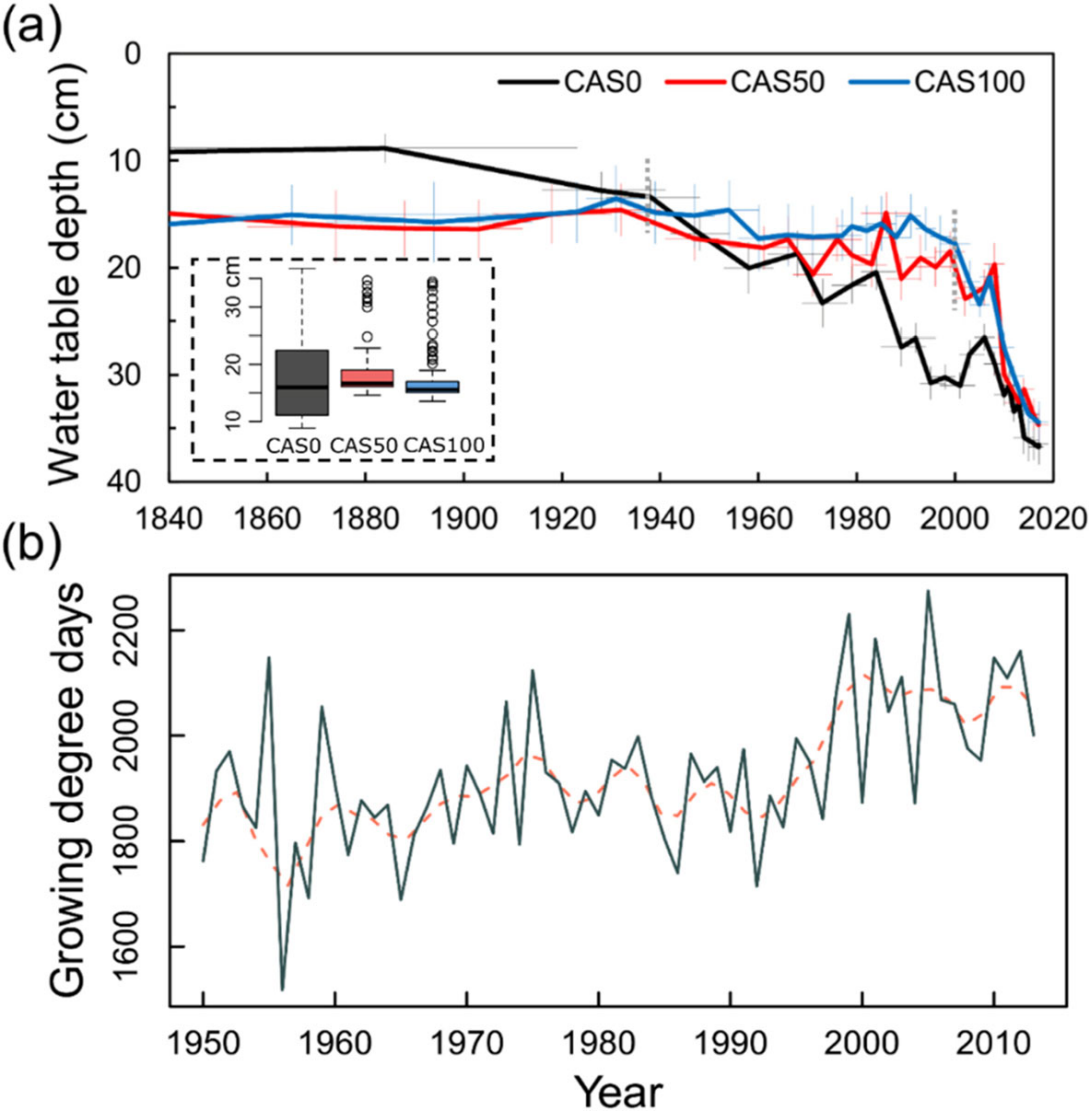
**a** WTD reconstructions for the post-fire period based on testate amoeba records and **b** growing degree days (> 0 °C) from May to September in the study area for the period 1950–2013. Error bars of both WTD reconstructions and age-depth modelling are shown by pale thin lines. Boxplots show the median, quartile range, extreme values (dotted lines), and outliers (dots) of the distribution. Tukey’s HSD test indicates no significant difference between the three sites. Grey dotted lines represent significant breakpoints (*P* <  0.01) in slopes based on Davies test. Climate data were extracted from McKenney et al. (2011). A 10-year loess smoothing is shown by the pink dashed line

Testate amoeba records indicate relatively wet conditions (high water table levels) shortly after the fire, followed by a gradual lowering of the water table at the three sites (Fig. 5a, Fig. S2.5). Inferred WTD values show very similar hydrological conditions at CAS50 and CAS100 during the post-fire period (1840–2017). Both sites had stable WTD between 15 and 20 cm before water tables deepened during the 2000s, particularly in the very recent horizons (~ 2010), while the water table lowered more gradually at CAS0. It remains unclear whether the apparent drying trend reflects increasingly drier surface conditions or simply an enhanced vertical growth of *Sphagnum* mosses that disconnected the peat surface from the water table. Indeed, warmer conditions observed since the 1990s (Fig. S2.6) have led to increasing growing degree days (Fig. 5b), which could have promoted this rapid peat accumulation.

## Discussion

### Stem growth decoupled from iWUE variations

Our study demonstrated that the paludification process considerably altered forest growth without influencing the intrinsic water use efficiency of black spruce trees. Sites with the thickest organic matter accumulation were characterized by dominant trees that grew slower, presented smaller heights and diameters (DBH), and had a lower tree density comparatively to the least paludified site (Table 1; Fig. 3). These results are in agreement with previous studies that documented forest productivity decline with increasing peat accumulation in the black spruce feather moss domain of eastern Canada (Harper et al. 2003; Fenton et al. 2005; Lecomte et al. 2006; Simard et al. 2007). Surprisingly, however, our tree-ring stable isotope analysis indicates no iWUE decrease over the last 100 years at the three study sites, but rather a substantial increase up to the 1980s (Fig. 4a).

The stable isotope analysis revealed that δ^13^C-derived parameters are not statistically different in all sites (Table 2), both in terms of average iWUE levels and temporal variations, suggesting that the ratio of assimilation rates to stomatal conductance is unaltered by the degree of paludification. We thereby also refute our second research hypothesis. In order to maintain comparable iWUE values across the paludification gradient (both in terms of mean and variability), the proportionality of the *A*/*g*_s_ ratio needs to be preserved between sites. This implies that if *A* is higher in the least paludified site (e.g., CAS0) and lower in the most paludified site (e.g., CAS100), then *g*_s_ will adjust in such a way to maintain nearly identical *A*/*g*_s_ ratio, and consequently, iWUE values. Actually, we suspect that this proportional adjustment in the *A*/*g*_s_ ratio might be an important process driving interactions between iWUE and growth rates in a paludified context. As a supporting evidence for this, we found that black spruce tree ring cellulose from the least paludified site (CAS0) was significantly more depleted in ^18^O compared to that of other sites (Fig. 4b). Unsurprisingly, CAS0 is also the site where radial growth rates are the highest. Increased evapotranspiration rates are probably required to sustain enhanced carbon assimilation and growth rates, forcing *g*_s_ to level up and proportionally adjust to increases in *A* (matching the *A*/*g*_s_ ratio of other sites). Consequently, higher evapotranspiration rates cause black spruce to pump more ^18^O-depleted water from soil depths (Evaristo et al. 2017), which in turn decreases the average δ^18^O of tree ring cellulose. Thus, the slower tree growth rates observed at the most paludified sites seem to result, at least partially, from both lower carbon assimilation rates (*A*) and stomatal conductance (*g*_s_).

These findings highlight that processes controlling radial tree growth are decoupled from those that control gas exchanges (*A* and *g*_s_), because of tree carbon use and allocation strategies. As an example, many studies performed in non-paludified forests have shown that iWUE increases do not directly translate into enhanced radial growth (e.g., Peñuelas et al. 2011; Lévesque et al. 2014; van der Sleen et al. 2015; Giguère-Croteau et al. 2019). Based on a comparison of tree ring widths and eddy-covariance flux towers in boreal Canada, Pappas et al. (2020) showed that aboveground biomass, and most particularly radial stem growth, represents only a minor fraction (~ 9%) of the total gross ecosystem production (GEP). Rocha et al. (2006) also found that stem growth, as estimated from tree ring widths, was not correlated to eddy-covariance-derived GEP in the boreal forest of central Manitoba. These findings point into the same direction: gas exchanges at the vegetation-atmosphere interface are controlled at the leaf level, but the allocation of newly formed photosynthates to either above or belowground compartments may depend on local growing conditions and site-specific growth strategies. Our study can therefore be seen as an extreme case where paludification induced locally-important edaphic changes, strong enough to impact tree carbon allocation strategies. Prioritization of belowground growth may have been more important in the most paludified sites, neglecting carbon allocation to aboveground compartments. This allocation strategy could reinforce tree anchoring (Nicoll et al. 2006) and enhance nutrient uptake (Vicca et al. 2012; Fernández-Martínez et al. 2014), but further research is needed to shed light on the processes driving allocation changes in black spruce trees.

### Synchronous changes in iWUE over time

From the 1920s and until the 1980s, a ~ 40% iWUE increase was observed at each site, regardless of the accumulated organic layer thickness. This important increase, which occurred over a short period of time, is among the highest recorded; most studies report iWUE increases of 20%–30% over the last century (e.g., Peñuelas et al. 2011; Silva and Horwath 2013; Saurer et al. 2014; Frank et al. 2015; van der Sleen et al. 2015). The increased iWUE resulted from an active response of trees characterized by the maintenance of a relatively constant *c*_i_ despite rising *c*_a_.

Nevertheless, in the 1980s, tree response to rising *c*_a_ suddenly became passive, as shown by the increasing *c*_i_ and the relatively constant iWUE values at all sites (Fig. 4a). Likewise, a shift to a passive response to increasing CO_2_ concentration has previously been observed for various tree species in Canada (Giguère-Croteau et al. 2019; Marchand et al. 2020), China (Wang et al. 2012; Wu et al. 2015), and Europe (Waterhouse et al. 2004; Gagen et al. 2011; Linares and Camarero 2012). Three reasons might explain this shift in acclimation strategies. Firstly, this finding possibly indicates reduced carbon assimilation rates (*A*). Indeed, in such poor growing environments, the photosynthesis apparatus may saturate, and nutrient limitation may downregulate the capacity of trees to assimilate atmospheric carbon (Tognetti et al. 2000; Saurer et al. 2003). Secondly, WTD reconstructions indicate important changes in hydrological conditions over the last 30 years (Fig. 5a) that might have altered black spruce iWUE. The recent water table drawdown could have generated stressful growth conditions since black spruce develops adventitious roots that are generally confined to the upper 20–30 cm of the organic layer (Lieffers and Rothwell 1987; Viereck and Johnston 1990). However, such a drop in WTD would have most certainly been accompanied by a reduction in stomatal conductance over time, which was not observed here. Moreover, the apparent drying trend could simply reflect an enhanced vertical *Sphagnum* moss growth, promoted by increasing growing degree days in the last decades (Fig. 5b; Magnan et al. 2018; van Bellen et al. 2018; Primeau and Garneau 2021; Robitaille et al. 2021). The rapid accumulation of organic matter may have exceeded the capacity of adventitious roots to develop higher in the soil profile, compromising the access to oxygen. Lastly, considering that black spruce trees were approximately 180 years old in the 1980s, we cannot rule out the stand age as another potential cause for the reduction in iWUE (Irvine et al. 2004; Kutsch et al. 2009; Marchand et al. 2020). Further studies are needed to determine which of these factors is mainly responsible for the significant shift observed in tree ecophysiological strategies in the 1980s.

### Ecophysiological adjustments to changing conditions

Changes in local environmental conditions have led to tree ecophysiological adaptations, particularly in the most paludified sites. However, these adjustments in growth mechanisms do not show in tree iWUE. The ratio *A* to *g*_s_ did not differ between sites, despite differences in peat thickness, water table depth, tree rooting depth, canopy cover, and tree growth rates (Tables 1 and 2; Fig. 3 and S2.2). These results indicate that iWUE variations do not reflect tree ecophysiological adjustments induced by changing growing conditions.

Changes in WTD over time (Fig. 5a) did not translate into diverging iWUE trends, in any of the sites. Although some important environmental factors were not investigated (e.g., nutrients, soil temperature), these results suggest that site-specific conditions were not determinant in iWUE variations. Instead, the synchronous shift in iWUE at each of the three sites in the mid-1980s is best explained by a response to region-wide changes in environmental conditions. Likewise, Pearson correlations calculated between standardized ring-width series and climate variables (monthly temperature and precipitation) indicate a shift in tree response to climate in the 1980s (Supplementary Material 1.1; Fig. S2.8). Black spruce trees became much less sensitive to both temperatures and precipitation after 1980. The reduced sensitivity of trees to temperature since the mid-twentieth century has been reported in previous tree ring studies of northern high-latitude forests, and has been referred to as the “divergence problem” (e.g., Briffa et al. 2004; D’Arrigo et al. 2008; Esper and Frank 2009; Schneider et al. 2014). This “divergence” phenomenon could potentially be caused by thresholded responses or stresses induced by changes in growth conditions (D’Arrigo et al. 2008). While further studies are required to shed light on such trends, these observations could support the hypothesis of photosynthesis apparatus saturation. Indeed, photosynthetic capacity, stimulated by elevated CO_2_ concentration, may have been limited by the poor growing conditions of forested peatlands, thus stabilizing iWUE and inhibiting stem growth response to temperature.

## Conclusions

In this study, we investigated the mechanisms that are driving tree growth decline in black-spruce-dominated forested peatlands of eastern Canada, by combining dendrochronological, paleoecological, and dendrogeochemical analyses. We attempted, for the first time, to unravel the numerous and complex entanglements between paludification dynamics and forest ecophysiology in these boreal ecosystems. Contrary to our expectations, tree growth decline induced by the paludification process does not result from a reduction of water use efficiency (iWUE). Indeed, we observed no significant differences in iWUE variations between the three study sites, which reflected different degrees of paludification. A substantial increase in iWUE was even recorded at each site for more than 50 years (1925 to 1985). Our tree-ring stable isotope analyses suggest that the decline in forest growth with increasing peat accumulation is rather explained by lower assimilation rates (*A*), together with lower stomatal conductance (*g*_s_), and possibly by the prioritization of carbon allocation to belowground components. Moreover, we found no evidence of tree ecophysiological adaptations to variations in water table depth. However, a significant shift in tree ecophysiology observed in the 1980s at all sites may suggest that the ratio between assimilation rates and stomatal conductance (iWUE) is influenced by regional or global factors, such as climate or increasing atmospheric CO_2_ concentration. These findings illustrate the complexity of the interactions between stem growth, ecophysiological processes, and environmental conditions, particularly in paludified sites. These dynamics will need to be further investigated to better predict the response of boreal forested peatlands to future climate change and improve forest management practices.

Our findings warrant further studies of vegetation/forest dynamics models and their application to forested peatlands, as those models are often biased towards converting increases in iWUE into increases in stem growth. Failing to account for paludification-related carbon use and allocation strategies would result in the overestimation of aboveground biomass production in sites where peat accumulation is substantial.

## Supplementary Information

**Additional file 1: Supplementary Method. Figure S2.1.** Photographs of the Casa forested peatland showing sites CAS100 (top) and CAS0 (bottom). **Figure S2.2.** Comparison of the three study sites in terms of peat accumulation, water table depth, tree height and diameter at breast height (DBH). Results of Tukey’s test indicate that CAS0 and CAS100 are significantly different for all of these parameters (*P*<0.01). Different letters above the boxes indicate significant differences between the sites based on Tukey’s test. **Figure S2.3**. Basal area increment distribution between the 25^th^ and the 75^th^ quantiles. Sites CAS0, CAS50, and CAS100 are shown in black, red, and blue respectively. The solid line represents the mean annual values of each site. **Figure S2.4**. Plant macrofossil diagrams of the three peat cores analysed. Data are presented in percentages (silhouettes) and in counts (bars, except for charcoals). The main peat components (%) are shown in the left column: *Sphagnum* (green), non-*Sphagnum* mosses (black), ligneous material (brown), Pteridophyte (orange), and Cyperaceae (white). The visual decay index, the dry density of peat, and the water table depth (WTD) reconstructions from testate amoeba assemblages (see figure S2.4) are also presented in the right columns. **Figure S2.5.** Testate amoeba diagrams. Diagrams show the abundance (%) of the dominant taxa in the three peat cores analysed. The water table depth (WTD) values inferred from testate amoeba records are presented in the right column (high values indicate dry conditions). Blanks in WTD reconstructions are due to exceptionally low test concentrations in some horizons, where the minimum count (20 tests) was not reached. The main peat components (%) are shown in the left column: *Sphagnum* (green), non-*Sphagnum* mosses (black), ligneous material (brown), Pteridophyte (orange), and Cyperaceae (white). **Figure S2.6.** March to September climate trends in the study area for the period 1950-2013. (a) precipitation, (b) temperature, and (c) growing degree days (>0°C). Linear trends are shown by dotted lines. Data were extracted from McKenney et al. (2011). **Figure S2.7.** Raw tree-ring-width series and standardized ring-width series. Sites CAS0, CAS50, and CAS100 are shown in black, red, and blue respectively. **Figure S2.8.** Pearson correlations between (a) standardized ring-width and monthly temperature, and (b) standardized ring-width and monthly precipitation for the periods 1950-1980 and 1981-2013. Correlation coefficients were calculated from March to September of the current year and the year preceding ring formation. Months from the previous year of stem growth are marked with an asterisk and significant correlations (*p* < 0.05) are marked with crosses. Results from CAS0, CAS50, and CAS100 are shown in black, red, and blue respectively. **Table S2.1**. Radiocarbon (^14^C) dates (Beaulne et al. 2021). **Supplementary References**

## Data Availability

Data will be archived on the Tree-Ring network of Qc-Lab database: http://dendro-qc-lab.ca.
