# Supplementary material for: Paludification reduces black spruce growth rate but does not alter tree water use efficiency in Canadian boreal forested peatlands
Source: For Ecosyst. 2021 May 12;8(1):28. doi: 10.1186/s40663-021-00307-x (PMC8550502; doi:10.1186/s40663-021-00307-x)
Supplement: Supplementary file 1 — Additional file 1: Supplementary Method. Figure S2.1. Photographs of the Casa forested peatland showing sites CAS100 (top) and CAS0 (bottom). Figure S2.2. Comparison of the three study sites in terms of peat accumulation, water table depth, tree height and diameter at breast height (DBH). Results of Tukey’s test indicate that CAS0 and CAS100 are significantly different for all of these parameters (P<0.01). Different letters above the boxes indicate significant differences between the sites based on Tukey’s test. Figure S2.3. Basal area increment distribution between the 25th and the 75th quantiles. Sites CAS0, CAS50, and CAS100 are shown in black, red, and blue respectively. The solid line represents the mean annual values of each site. Figure S2.4. Plant macrofossil diagrams of the three peat cores analysed. Data are presented in percentages (silhouettes) and in counts (bars, except for charcoals). The main peat components (%) are shown in the left column: Sphagnum (green), non-Sphagnum mosses (black), ligneous material (brown), Pteridophyte (orange), and Cyperaceae (white). The visual decay index, the dry density of peat, and the water table depth (WTD) reconstructions from testate amoeba assemblages (see figure S2.4) are also presented in the right columns. Figure S2.5. Testate amoeba diagrams. Diagrams show the abundance (%) of the dominant taxa in the three peat cores analysed. The water table depth (WTD) values inferred from testate amoeba records are presented in the right column (high values indicate dry conditions). Blanks in WTD reconstructions are due to exceptionally low test concentrations in some horizons, where the minimum count (20 tests) was not reached. The main peat components (%) are shown in the left column: Sphagnum (green), non-Sphagnum mosses (black), ligneous material (brown), Pteridophyte (orange), and Cyperaceae (white). Figure S2.6. March to September climate trends in the study area for the period 1950-2013. (a) precipitation, (b) temper [file 40663_2021_307_MOESM1_ESM.pdf]

Paludification reduces black spruce growth rate but does not alter tree water use efficiency in Canadian boreal forested peatlands

Joannie Beaulne[1,2,3*], Étienne Boucher[1,2,4], Michelle Garneau[1,2,3,4], and Gabriel Magnan[1,2,3]

[1] Geotop Research Center, Université du Québec à Montréal, Montréal, Québec H3C 3P8, Canada

[2] Department of Geography, Université du Québec à Montréal, Montréal, Québec H3C 3P8, Canada

[3] GRIL-UQAM, Université du Québec à Montréal, Montréal, Québec H3C 3P8, Canada

[4] Centre d'études nordiques, Université Laval, Québec, Québec G1V 0A6, Canada

* Email: joannie.beaulne@gmail.com

Supplementary material

1. Supplementary methods

1.1 Tree rings and climate analysis

Ring-width series were standardized using a negative exponential curve to remove cambial age trends (Fritts 1976). Standardization was performed on all individual series before constructing a mean standardized chronology for each site. Daily climate data (mean temperature and total precipitation) from 1950 to 2013 were retrieved from the interpolated gridded climate dataset of McKenney et al. (2011). Pearson correlation coefficients were calculated between standardized ring-width series and monthly climate data from March to September of both the current year and the year preceding ring formation. Because of time series autocorrelation, effective numbers of degrees of freedom were calculated to generate adjusted *p*-values (Hu et al. 2017).

1.2 Isotopic analysis of tree rings

Black spruce ecophysiological mechanisms were investigated through $\delta^{13}C$ and $\delta^{18}O$ analyses. These analyses were performed on cross-sections from five trees per site. For each selected cross-section, two wood strips of 3 × 10 mm were cut and finely sanded on all sides. A five-year resolution covering a 100-year period (1919-2018) was considered. After having carefully cross-dated the wood strips, tree rings were cut with a razor blade under a binocular microscope. Rings of the same years were pooled together in equal amounts, resulting in 20 subsamples of five years per site, which were then grinded using a mixer mill (Retsch MM400) to ensure homogeneity (Borella et al. 1998). Alpha-cellulose was then extracted as suggested for black spruce samples (Bégin et al. 2015) following the protocol used by Naulier et al. (2014).

All isotopic analyses were carried out at the Light stable isotope geochemistry laboratory of the GEOTOP Research Center (Université du Québec à Montréal, Canada). Stable isotope ratios of

carbon and oxygen were analyzed with an isotope ratio mass spectrometer (Isoprime 100 for $\delta^{13}C$ and Isoprime VisIon for $\delta^{18}O$) coupled to an elemental analyser (Elementar Vario MicroCube for $\delta^{13}C$ and Elementar Vario PyroCube for $\delta^{18}O$) in continuous flow mode. Results were normalized with three internal standards on NBS19-LSVEC and VSMOW-SLAP scales for $\delta^{13}C$ and $\delta^{18}O$ respectively. Results are reported in ‰ (± 0.1‰ for $\delta^{13}C$ and ± 0.3‰ for $\delta^{18}O$) relative to VPDB for carbon isotopic ratios and to VSMOW for oxygen isotopic ratios. Because of the high combustion of ^{13}C-depleted fossil fuels since the industrial period (~1850 CE), atmospheric $^{13}CO_2$ concentration is significantly decreasing, which causes a declining trend in tree ring $\delta^{13}C$ values. This Suess effect was therefore corrected as proposed by McCarroll and Loader (2004).

1.3 Testate amoeba analysis

Testate amoeba shells were extracted from 1 cm^3 peat subsamples following the standard protocol of Booth et al. (2010). Subsamples were gently boiled in distilled water and washed through 300 and 15 µm sieves. The material remaining in the 15 µm mesh was stained and mounted on glass slides before being analysed under an optical microscope (400 × magnification). Testate amoebae were identified following the taxonomy of Mitchell (2002), Siemensma (2018), and Charman et al. (2000), with the modifications of Booth and Sullivan (2007).

The dataset of the transfer function used to reconstruct WTD variations (Lamarre et al. 2013) was improved by adding 40 surface samples collected along the study transect, and 40 others sampled in another forested peatland in the study area. Peat surface samples of approximately 10 cm^3 were cut with a serrated knife, following the method described in Lamarre et al. (2013). Samples were collected in lawns and hummocks, as these are the only microforms found in our sites.

1.4 Macrofossil analysis

Subsamples of 4 cm^3 were gently boiled in a 5% KOH solution before being washed through a 125 µm mesh sieve, following the protocol of Mauquoy et al. (2010). Macrofossils were analysed in a gridded Petri dish under a stereomicroscope (10-40 × magnification), using Lévesque et al. (1988) and Mauquoy and van Geel (2007) for plant identification. The relative abundances of the main peat components (e.g., *Sphagnum*, ligneous, Cyperaceae) were estimated visually and expressed as volume percentages, and vascular plant remains (e.g., seeds, needles, leaves) were counted. The degree of plant material decomposition was also determined visually using an index ranging from 1 (poorly decomposed) to 5 (highly decomposed). Macroscopic charcoal particles (>0.5 mm) were analysed at 1 cm intervals along the three peat cores to identify past local fire events (Beaulne et al. 2021).

2. Supplementary figures and tables

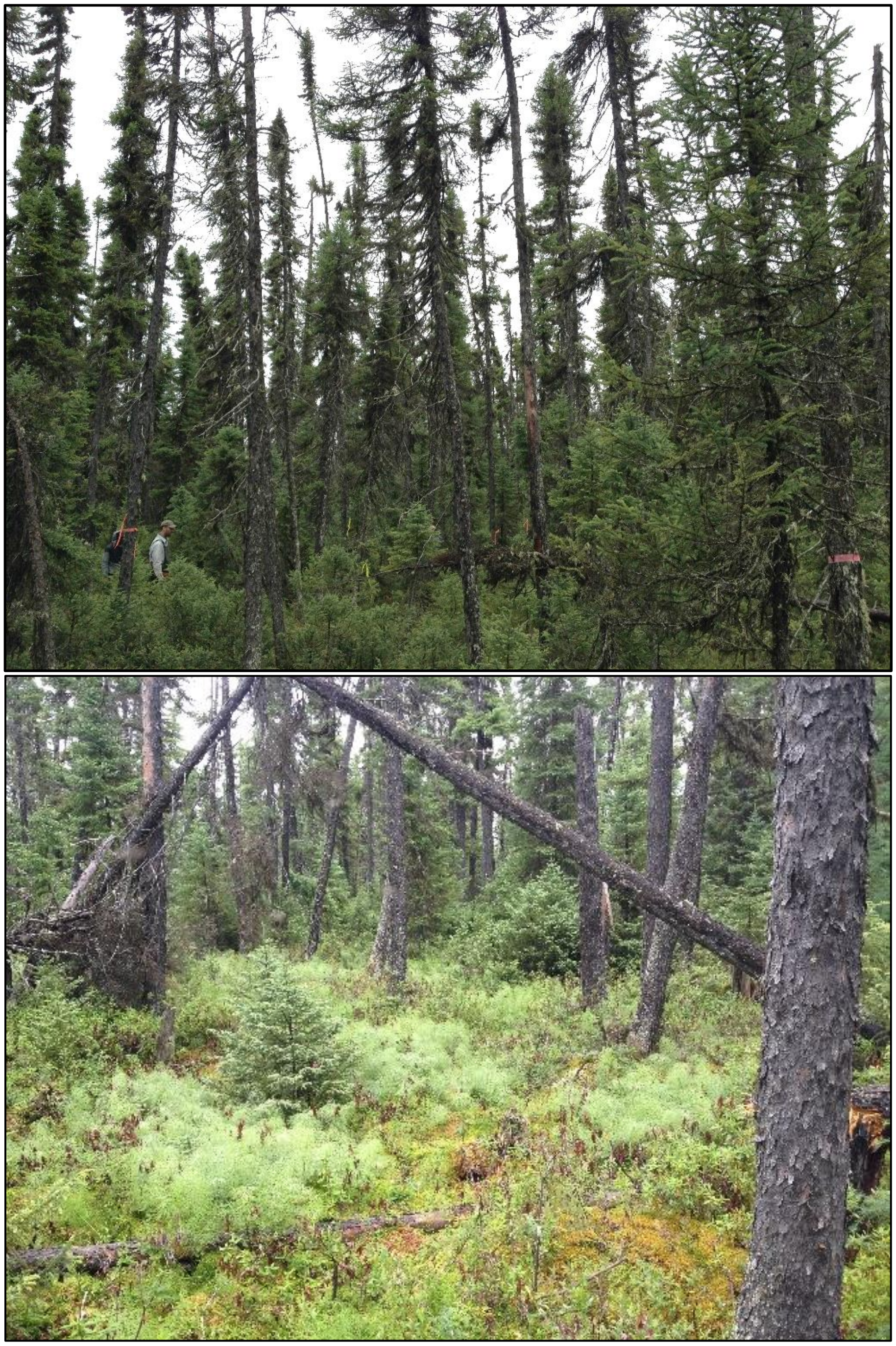

Figure S2.1. Photographs of the Casa forested peatland showing sites CAS100 (top) and CAS0 (bottom).

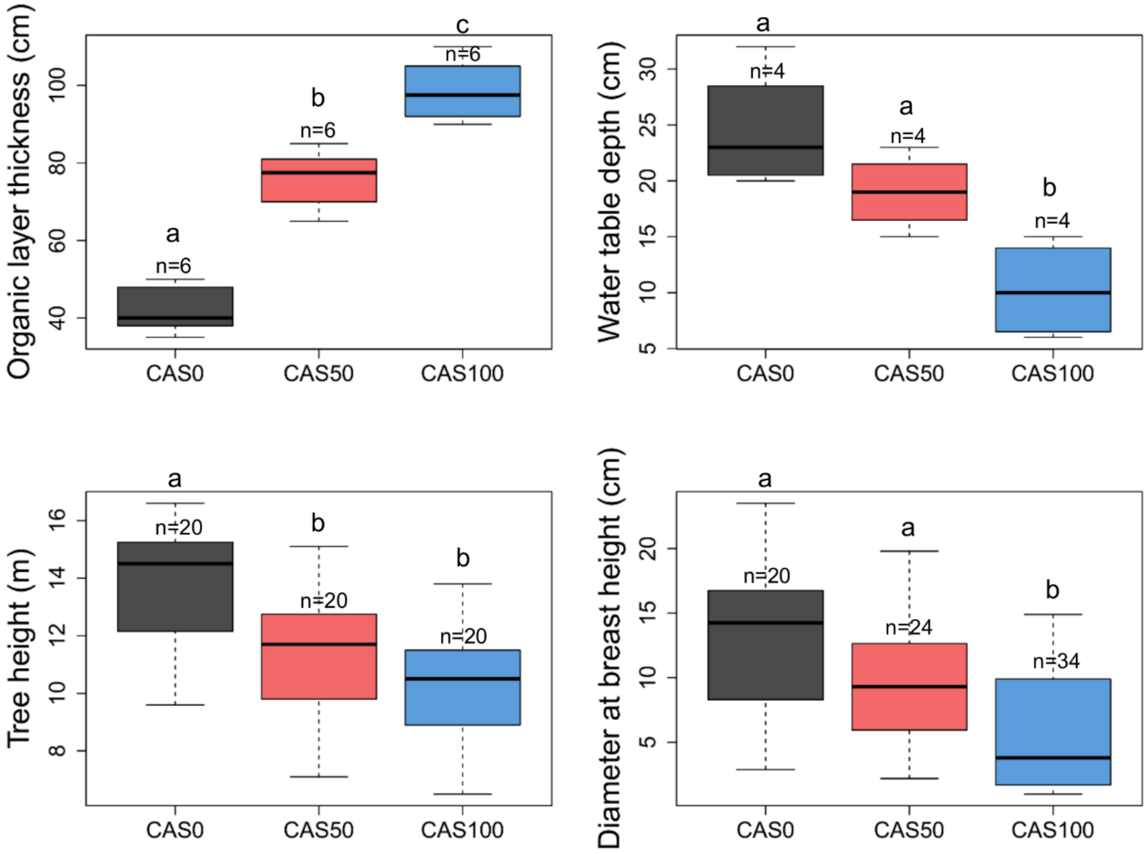

Figure S2.2. Comparison of the three study sites in terms of peat accumulation, water table depth, tree height and diameter at breast height (DBH). Results of Tukey's test indicate that CAS0 and CAS100 are significantly different for all of these parameters (P<0.01). Different letters above the boxes indicate significant differences between the sites based on Tukey's test.

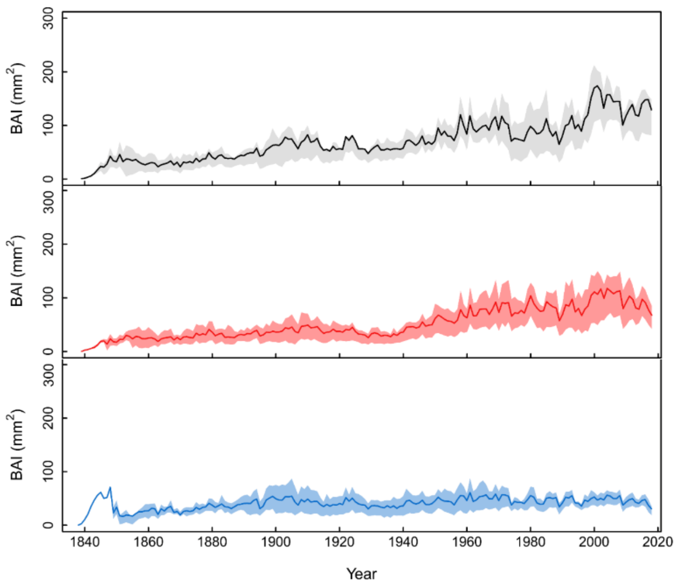

Figure S2.3. Basal area increment distribution between the 25th and the 75th quantiles. Sites CAS0, CAS50, and CAS100 are shown in black, red, and blue respectively. The solid line represents the mean annual values of each site.

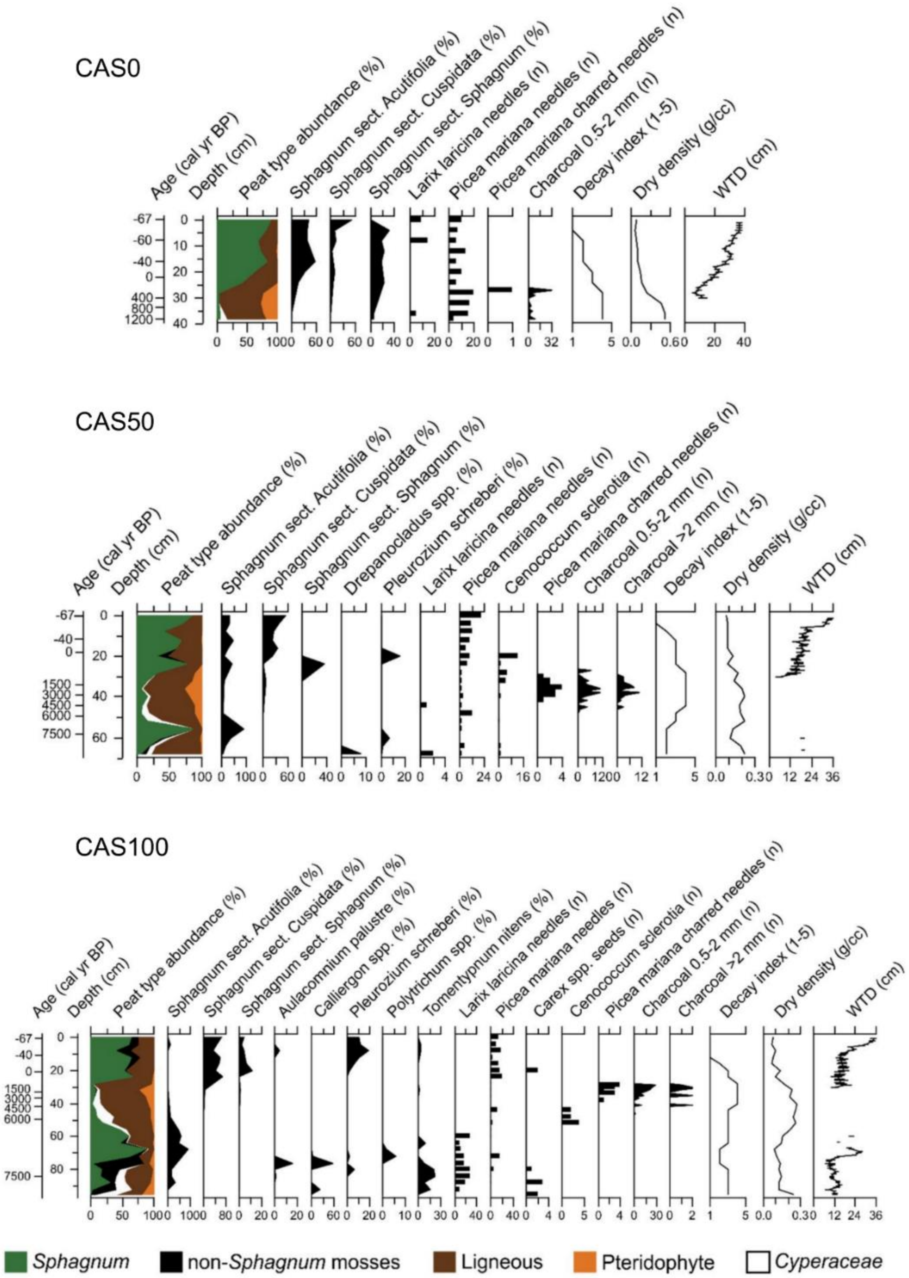

Figure S2.4. Plant macrofossil diagrams of the three peat cores analysed. Data are presented in percentages (silhouettes) and in counts (bars, except for charcoals). The main peat components (%) are shown in the left column: *Sphagnum* (green), non-*Sphagnum* mosses (black), ligneous material (brown), Pteridophyte (orange), and Cyperaceae (white). The visual decay index, the dry density of peat, and the water table depth (WTD) reconstructions from testate amoeba assemblages (see figure S2.4) are also presented in the right columns.

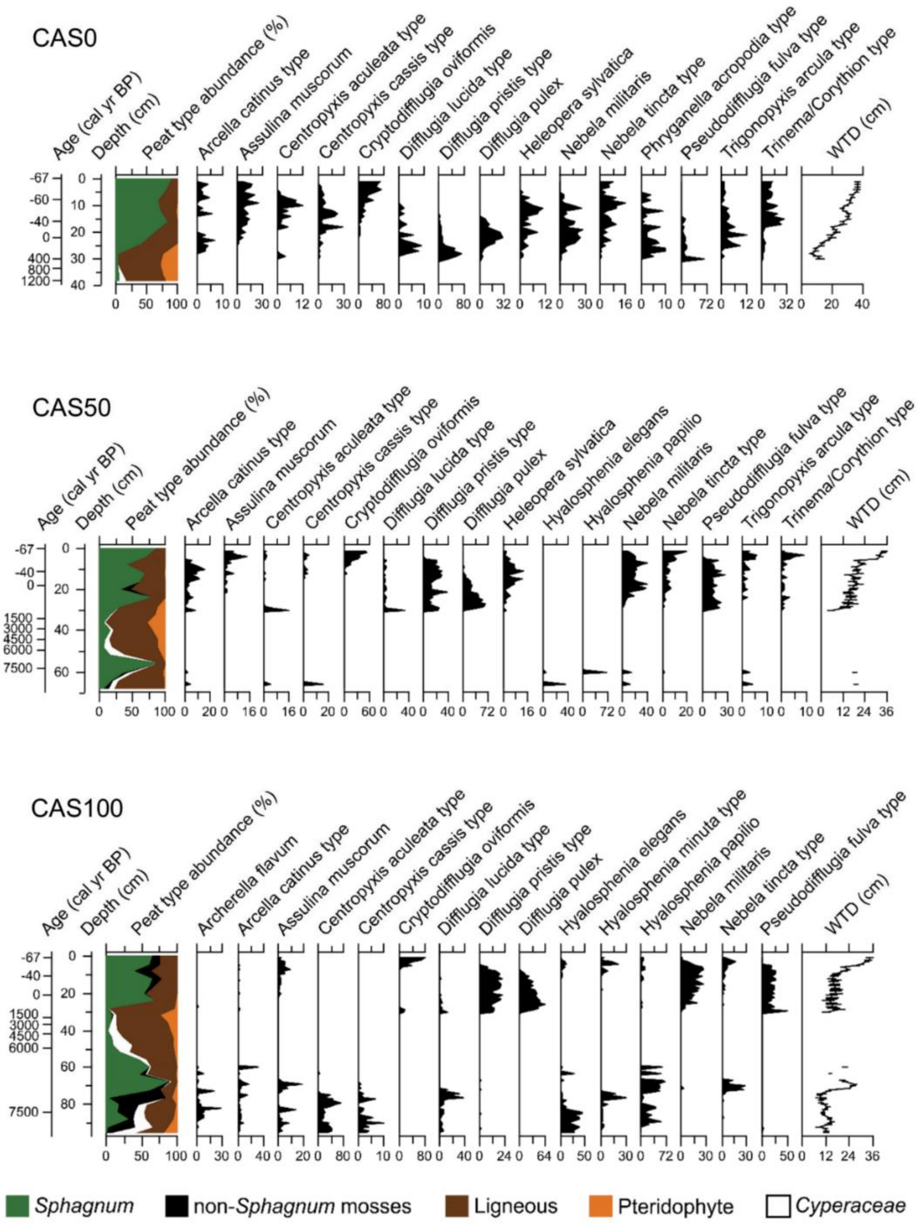

Figure S2.5. Testate amoeba diagrams. Diagrams show the abundance (%) of the dominant taxa in the three peat cores analysed. The water table depth (WTD) values inferred from testate amoeba records are presented in the right column (high values indicate dry conditions). Blanks in WTD reconstructions are due to exceptionally low test concentrations in some horizons, where the minimum count (20 tests) was not reached. The main peat components (%) are shown in the left column: *Sphagnum* (green), non-*Sphagnum* mosses (black), ligneous material (brown), Pteridophyte (orange), and Cyperaceae (white).

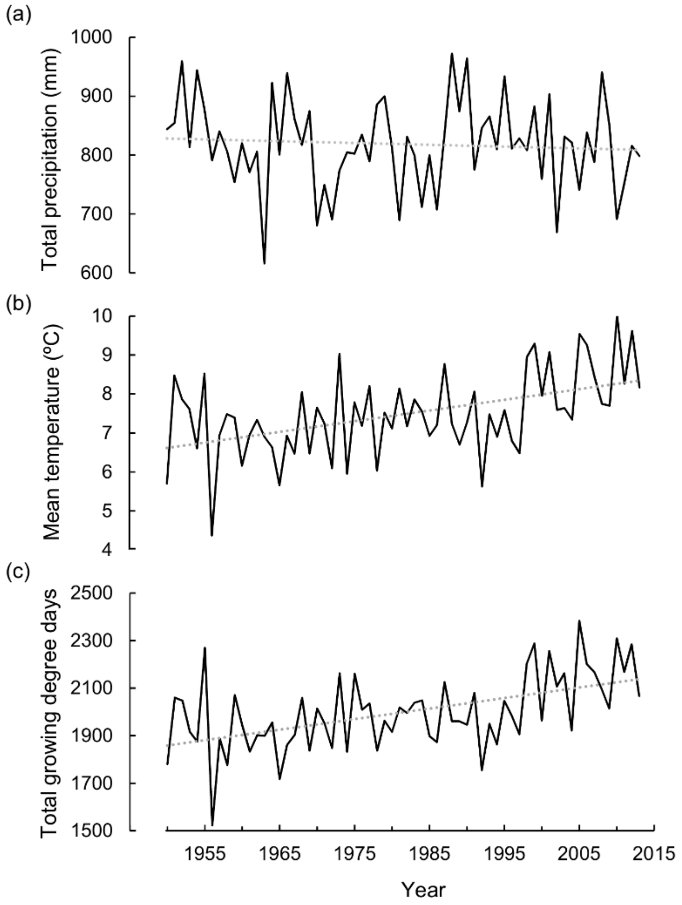

Figure S2.6. March to September climate trends in the study area for the period 1950-2013. (a) precipitation, (b) temperature, and (c) growing degree days (>0°C). Linear trends are shown by dotted lines. Data were extracted from McKenney et al. (2011).

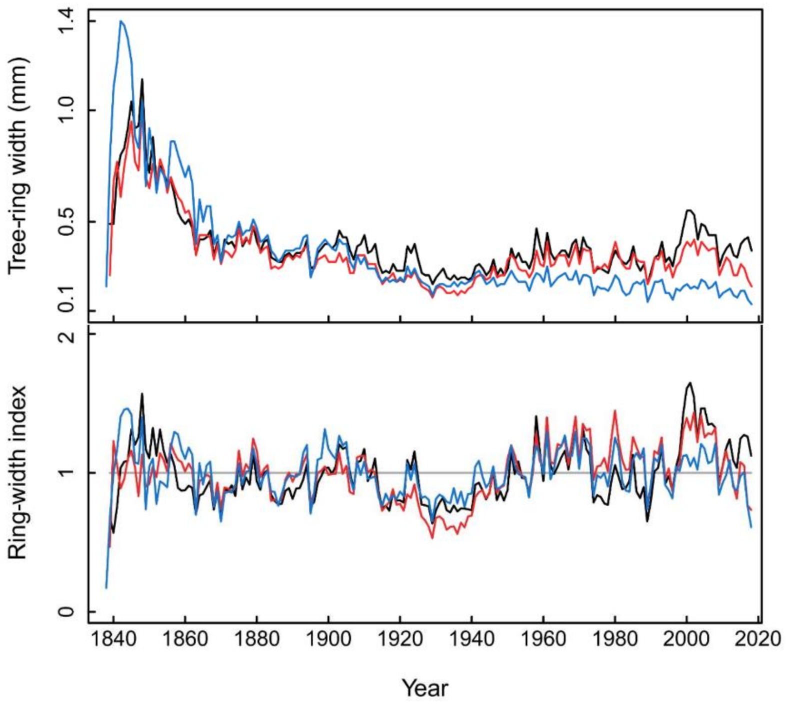

Figure S2.7. Raw tree-ring-width series and standardized ring-width series. Sites CAS0, CAS50, and CAS100 are shown in black, red, and blue respectively.

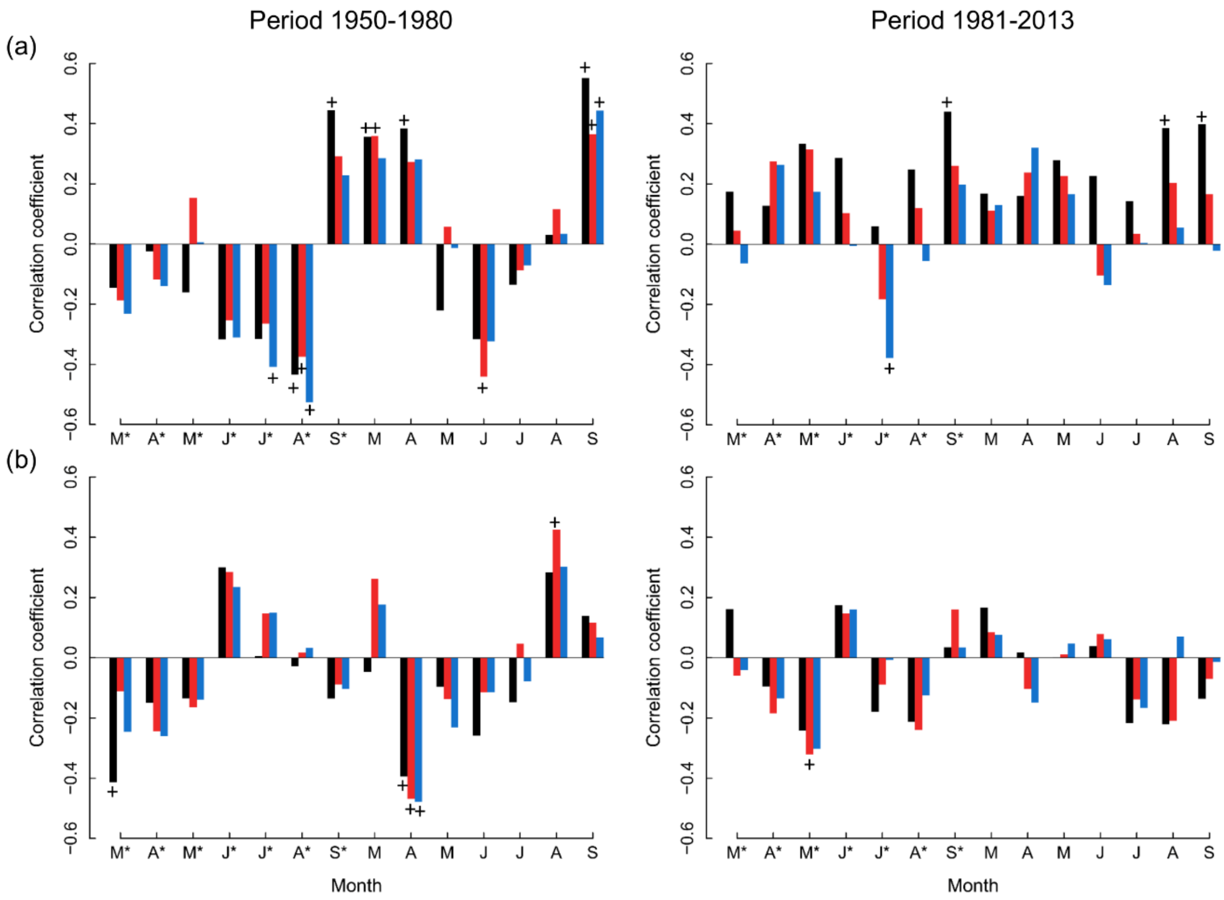

Figure S2.8. Pearson correlations between (a) standardized ring-width and monthly temperature, and (b) standardized ring-width and monthly precipitation for the periods 1950-1980 and 1981-2013. Correlation coefficients were calculated from March to September of the current year and the year preceding ring formation. Months from the previous year of stem growth are marked with an asterisk and significant correlations ($p < 0.05$) are marked with crosses. Results from CAS0, CAS50, and CAS100 are shown in black, red, and blue respectively.

Table S2.1. Radiocarbon (^{14}C) dates (Beaulne et al. 2021).

Core	Depth (cm)	Laboratory number	Material dated	Radiocarbon age ± error (^{14}C yr BP)	Calibrated age range (cal yr BP)	Median age (cal yr BP)
CAS0	26-27	UOC-9614	Charcoal, charred needles	158±29	0-285	175
	38-39	UOC-8508	Bulk peat	1314±32	1182-1296	1255
CAS50	26-27	UOC-9615	Charcoal, charred needles	171±29	0-290	179
	33-34	UOC-8509	Charcoal, charred needles	1264±24	1175-1281	1224
	56-57	UOC-9617	*Sphagnum* stems	6610±29	7440-7565	7502
	69-70	UOC-9616	Bulk peat	6838±29	7610-7724	7667
CAS100	28-29	UOC-8512	Charcoal, charred needles	154±24	35-284	178
	44-45	UOC-8513	*Picea* needles, *Sphagnum* stems	4691±31	5320-5577	5397
	52-53	UOC-8514	*Picea* needles, *Sphagnum* stems	5469±38	6190-6386	6274
	72-73	UOC-8515	*Picea* and *Larix* needles	6466±34	7311-7436	7374
	95-96	UOC-6053	Bulk peat	6635±41	7441-7578	7522

3. Supplementary references

Beaulne J, Garneau M, Magnan G, Boucher É (2021) Peat deposits store more carbon than trees in forested peatlands of the boreal biome. Sci Rep 11:2657.

Bégin C, Gingras M, Savard MM, Marion J, Nicault A, Bégin Y (2015) Assessing tree-ring carbon and oxygen stable isotopes for climate reconstruction in the Canadian northeastern boreal forest. Palaeogeogr Palaeoclimatol Palaeoecol 423:91-101.

Booth RK, Lamentowicz M, Charman DJ (2010) Preparation and analysis of testate amoebae in peatland palaeoenvironmental studies. Mires Peat 7:1-7.

Booth RK, Sullivan M (2007) Key to testate amoebae inhabiting Sphagnum-dominated peatlands with an emphasis on taxa preserved in Holocene sediments. Earth and Environmental Science Department, Lehigh University, Bethlehem.

Borella S, Leuenberger M, Saurer M, Siegwolf R (1998) Reducing uncertainties in $\delta^{13}C$ analysis of tree rings: pooling, milling and cellulose extraction. J Geophys Res 103:19519-19526.

Charman DJ, Hendon D, Woodland WA (2000) The identification of Testate Amoebae (Protozoa: Rhizopoda) in peats, Technical Guide No. 9. Quaternary Research Association, London.

Fritts HC (1976) Tree rings and climate. Academic Press, New York.

Hu J, Emile-Geay J, Pardin J (2017) Correlation-based interpretations of paleoclimate data – where statistics meet past climates. Earth Planet Sci Lett 459:362-371.

Lamarre A, Magnan G, Garneau M, Boucher É (2013) A testate amoeba-based transfer function for paleohydrological reconstruction from boreal and subarctic peatlands in northeastern Canada. *Quat Int* 306:88-96.

Lévesque PEM, Dinel H, Larouche A (1988) Guide illustré des macrofossiles végétaux des tourbières du Canada. Agriculture du Canada, Direction générale de la recherche. Publication no. 1817.

Mauquoy D, Hughes PDM, van Geel B (2010) A protocol for plant macrofossil analysis of peat deposits. Mires Peat 7:1-5.

Mauquoy D, van Geel B (2007) Plant macrofossil methods and studies: Mire and peat macros. In SA Elias (Ed.) Encyclopedia of Quaternary Science. Elsevier Science, Amsterdam.

McCarroll D, Loader NJ (2004) Stable isotopes in tree rings. Quat Sci Rev 23:771-801.

McKenney DW, Hutchinson MF, Papadopol P, Lawrence K, Pedlar J, Campbell K, Milewska E, Hopkinson RF, Price D, Owen T (2011) Customized spatial climate models for North America. Bull Am Meteorol Soc December 1612-1622.

Mitchell EAD (2002) The identification of *Centropyxis*, *Cyclopyxis*, *Trigonopyxis* and similar *Phryganella* species living in *Sphagnum*. Available online at http://istar.wikidot.com/id-keys.

Naulier N, Savard MM, Bégin C, Marion J, Arseneault D, Bégin Y (2014) Carbon and oxygen isotopes of lakeshore black spruce trees in northeastern Canada as proxies for climatic reconstruction. Chem Geol 374-375:7-43.

Siemensma F (2018) Microworld – World of amoeboid organisms. Available online at http://www.arcella.nl/.
